# Results of the cementless Plasmacup in revision total hip arthroplasty: a retrospective study of 72 cases with an average follow-up of eight years

**DOI:** 10.1186/1471-2474-11-101

**Published:** 2010-05-27

**Authors:** Stefan Lakemeier, Guenter Aurand, Nina Timmesfeld, Thomas J Heyse, Susanne Fuchs-Winkelmann, Markus D Schofer

**Affiliations:** 1 Department of Orthopaedics and Rheumathology, Baldingerstraße, 35043 Marburg, University Hospital Gießen and Marburg, location Marburg, Germany; 2 Institute of Medical Biometry and Epidemiology, Bunsenstraße 3, 35037 Marburg, Phillips-University Marburg, Germany

## Abstract

**Background:**

There are multiple revision implant systems currently available for socket revision in revision total hip arthroplasty. Up until now, not all of these systems have been followed up with regards to their long-term use as a revision implantation.

For the first time, this study presents the hemispherical porous-coated socket Plasmacup SC, produced by Aesculap, Tuttlingen, Germany, and the clinical and radiological mid-term results of this revision cup implant.

**Methods:**

Over a period of ten years the Plasmacup SC press-fit-cup was used as a revision implant in 72 consecutive aseptic cases which were included in this retrospective study. The mean follow-up period was 8 years. Bone graft transplantation was performed in 32% of all cases. In 90%, the cup was fixed with additional screws. The follow-up radiographs were analysed with regards to cup migration, osteointegration and osteolysis in the DeLee zones using a computer aided program taking the teardrop figure as a main point of reference. For clinical evaluation the Harris-Hip-Score and the WOMAC-Score were utilized.

**Results:**

At the follow up examination, the mean Harris-Hip-Score was 83.5 points and the mean WOMAC-Score 34.7 points. 93% of all patients were satisfied with the result of the operation. No aseptic cup loosening could be observed and only one cup had to be removed due to infection. No significant longitudinal or transversal cup migration could be observed.

**Conclusion:**

Aesculap's Plasmacup SC is suitable as a cementless cup revision implant. There is stable cup osteointegration, post press-fit implantation, even in the case of major acetabular bone defects.

## Background

The significance of revision total hip arthroplasty is continuously increasing. While in 2003 the ratio of primary endoprosthesis to revision surgery was approximately 1:14, it was stated as 1:7 in 2006 [1]. In a study published recently, based on data obtained from the Finnish arthroplasty register, similar long-term survival rates were described for cemented and cementless THA in patients aged more than 55 years [2]. Whereas aseptic loosening is the most common reason for revision of cemented cups, polyethylene wear and osteolysis are mainly responsible for revision of cementless cups [3]. The acetabular component is affected twice as frequently as the stem [4]. The aim of socket revision surgery is the permanent and solid fixation of the new socket, the reconstruction of the acetabular bone stock and the correct rebuilding of the hip's centre of rotation. For implant revisions there is a great variety of models using cementless or cemented fixation techniques. Cementing a cup into the existing defect often provides bad results in the case of revision. Engelbrecht et al. report 29% of loosening after 8 years [5]. The results of cemented cups in revision THA can be improved using impaction bone grafting. The advantages of this method include the ability to restore bone stock, rebuild normal hip center and hip biomechanics, and increase bone stock for future revisions [6]. Sembrano and Cheng described acceptable results with five-year loosening-free and acetabular reoperation-free survivorships of 80.7% after application of trabecular metal acetabular cages as acetabular revision implants [7]. One the one hand this procedure is complex but on the other hand treatment even of major bone defects is possible [8]. There are different results after the implantation of a cementless oblong revision cup. Koster et al. noticed 2% of aseptic loosening after 3.6 years, Götze et al. 12% after an average of 2.8 years [9,10].

Another possibility is the implantation of a cementless hemispheric press-fit-cup with the option of additional screw fixation. The applicability of this type of socket has been well documented for some models [11-13]. One significant advantage is that the implantation technique is less complex. The question if bone graft transplantation is necessary in order to achieve good long-term results is still point of discussion. Parratte et al. found good results using hemispherical press-fit cups with morselized bone graft for both, the restoration of the acetabular bone stock and the stabilization of the cup [14].

Other authors define the position that hemispherical sockets can only achieve long-term implant fixation in acetabular defects that are not extensive. Christie points out that in order to achieve intimate contact between implant and host bone, which is critical for stability since bone ingrowth requires complete absence of micromotion, the implant must match the defect or be able to bridge it. For this reason, increasing bone loss requires the use of other revision implants such as a hooked roof cup or an oblong cup [15]. Although the design of the Plasmacup SC (Aesculap, Tuttlingen, Germany) is similar to other press-fit cups, the Plasmacup is provided with a special rough titanium micro-porous coating with smaller pore size (50 - 200 μm) compared to other press-fit cups. Because of the rough surface und the osteoconductivity of the titanium coating higher primary and secondary stability is expected [16].

The aim of the present retrospective study was to describe the clinical and radiological results of the Plasmacup SC in order to show the applicability of this devise as revision implant in revision THA.

## Methods

72 socket revisions were carried out from 01 January 1998 until 31 December 2007, using the cementless press-fit-cup Plasmacup SC, produced by Aesculap, Tuttlingen, Germany.

Surgery was performed on 69 patients, whereupon 3 patients were operated on both sides. At the time of surgery, the average age of the patients was 65.4 (43 - 81). The reason for the revision surgery was an aseptic loosening of the socket. 47 cemented cups and 25 cementless cups were revised. 31 socket revision procedures were performed carrying out stem revisions. Bony defects were classified by an independent examiner according to Paprosky on the basis of the operative reports and using preoperative radiographs (a.p. and oblique views) [17]. The decision whether and to which extent the autogenous or allogenic bone grafting was necessary, was decided by the surgeon during surgery. 95% of the operations were carried out by one surgeon, 3% by a second and 2% by a third one. Bauer's transgluteal approach was chosen in all cases and the cementless press-fit-cup, the concept of which was thoroughly researched and described by Stalforth et al. in 1998, was implanted [18]. The average size of the implanted sockets in men was 60.1 (52 - 66) mm and in women 56.3 (46 - 66) mm. The cup diameter exceeded that of the explanted sockets by an average of 7.3 (4 - 10) mm.

### Patient follow-up examination

Full ethical approval was granted for the project by the local ethics committee. Preoperative informed consent was obtained in all cases prior to the inclusion into this study.

The mean observation period was 97 (5 - 120) months. 58 patients had a minimum follow-up of 24 month. Out of 72 socket revisions performed on 69 patients, 68 socket revisions performed on 66 patients could be entered into the study. 4 socket revisions performed on 3 patients could not be entered into the study. Due to an early infection, one socket had to be explanted post operatively 35 days after surgery. No data collection could be carried out on 2 further patients who received 3 socket revisions because the patients moved to an unknown address and no information was obtainable about their postoperative course.

Out of the 66 patients, 55 were followed up within the framework of study. 11 patients were deceased at the time of the follow-up examination. For possible future studies, we routinely collect the data for the Harris-Hip-Score and the WOMAC-Score ((Western-Ontario and Mac Masters University Score) for our patients treated with revision total hip arthroplasty in each check-up examination. For the deceased patients the data for this study was taken from their last check-up. The deaths were not connected to the socket revisions.

For all other patients the Harris-Hip-Score and the WOMAC-Score were collected at the follow-up examination by an independent examiner (orthopedics specialist) [19,20]. The Harris-Hip-Score result was assessed as "very good" with a score of 90 - 100 points, as "good" with 80 - 89 points and as "satisfactory" with 70 - 79 points. Point scores below 70 showed a bad result. The WOMAC-Score examined the areas "pain", "stiffness" and "physical activity". The maximum points achievable were 240. A high score indicated a bad clinical result.

All patients were asked whether and to what extent they were still taking pain killers due to hip pain at the time of examination. For pain quantization, all participants assessed their existing hip pain using the "visual analogue scale" (VAS) and points scores between 0 (no pain) and 10 (strongest pain) [21].

### Radiograph analysis

All available radiographs were divided into 6 groups in order to obtain sufficiently sized sets:

- postoperative (all photographs up to postoperative day 42)

- 0.5 years (all photographs from postoperative day 43 to 9 months post operation)

- 1 year (all photographs starting 10 months post-operation to 1.75 years post-operation)

- 2-3 years (all photographs from 1.76 to 3.5 years post operation)

- 4-5 years (all photographs from 3.6 to 5.5 years post operation)

- more than 6 years (all photographs older than 5.5 years)

The classification was necessary because the check-up examinations did not always occur at regular intervals and radiographs were not always available for every patient at the time of every follow-up examination.

Thus, 336 radiographs were entered retrospectively into the study. The processing of the photographs was done digitally after scanning the radiographs using a film digitizer VXR-12 (Vidar Systems Corporation, Herndon, Virginia, USA) digital and the "Wristing" programme. This programme was first introduced by Bach et al. in 2005 and was validated for the digital measurement of radiographs [22]. It uses the bottom edge of the tear drop figure as point of reference. In order to determine the cup migration, the following four distances were observed using the "Wristing" programme:

- Top edge of cup - tear figure

- Medial edge of cup - tear figure

- Cup centre - tear figure longitudinal

- Cup centre - tear figure transversal

When evaluating the postoperative results, the following radiological findings were taken as an indication for cup loosening [23,24]:

- a circumferential zone pervious to radiographs of more than 2 mm

- cup migration of more than 3 mm

- change of inclination of more than 8 degrees

Osteolyses and radiolucent lines were determined in the zones defined by DeLee and Charnley by dividing the contact area from cup to bone into three segments.

Radiolucency was classified according to position, size and progression [25].

### Statistics

The measurements for the cups' individual movement directions were evaluated using a mixed linear model. As an accidental effect, the individual patient measurements were modelled. The basis for this was the immediate postoperative reading. For the observation of the change in position over the entire period, a variance analysis (F-test) was applied. All available radiographs were used for the adaptation of the model. The evaluation was carried out using the statistics programme "R" of the R-Foundation for Statistical Computing, Vienna, Austria. The significance level was set at 5%. A normal distribution of the measured data was assumed for the calculation.

## Results

The average Harris-Hip-Score was 83.5 (9 - 100) points during the follow-up examination receiving a corresponding assessment of "good". The mean value for the category "pain" was 41.5 (0 - 44) points, and hip function had an average of 34 (0 - 47) points. The Harris-Hip-Score result was assessed as "very good" in 45% of the cases; 21% were "good" and 23% of patients had a "satisfactory" result. In 11% of the cases, the result was "bad".

The mean WOMAC Score was 37.4 (0 - 204) points.

Patients quoted pain in the operated hip joint with an average of 1.3 (0 - 7) on the VAS.

4 patients (5%) took pain killers regularly at the time of the last follow-up examination due to discomfort in the hip joint operated on. These patients stated an average value of 4.8 (4 - 7) on the VAS. 7% of all patients had a positive "sign of Trendelenburg". No patient showed symptoms of anterior psoas irritation.

All cups included in the follow-up examination were in place at that time.

### Radiograph analysis

The average distance between the top edge of the cup and the tear figure changed only very slightly during the follow-up examination period of more than 6 years (additional file 1). The differences of up to 0.75 mm in the measurement data fall within the measurement limits of inaccuracy (additional file 1).

No migration in longitudinal direction could be ascertained during the follow-up examination period.

No significant change of position in transversal direction could be detected. The distances between the medial cup edge and the tear figure, if anything, became smaller. The comparison of the distances from the tear figure to the centre of the cup in transversal direction shows no statistically relevant movement of the cup's edge towards the medial. All observed differences are within the margin of error of measurement for the procedure.

The comparison of cup inclination shows an increase in inclination (p < 0.0001) of an average of 3.4° between the postoperative radiograph and that taken after more than 6 years. The anteversion showed no significant change in position over the entire period (additional file 1).

In summary, it can be stated that a statistically relevant change in position of the implanted cups only exists with regards to the increase of 3.4° in cup inclination between the radiograph taken directly post-operation and the one taken after 6 years. A large part of this change in position took place between the time directly post-operation and 1 year post-operation. The exact data are shown in additional file 1.

**Figure 1:**
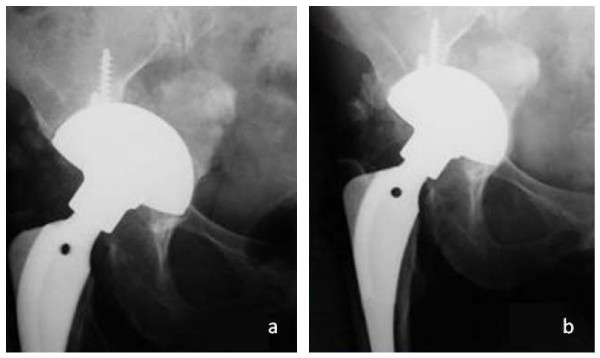
**Radiolucent lines in DeLee zone 1 and 3 directly after cup implantation (Plasmacup SC, size 60 mm) with bone graft transplantation (a)**. Complete regression after three years postoperative and firm cup incorporation (b).

**Figure 2:**
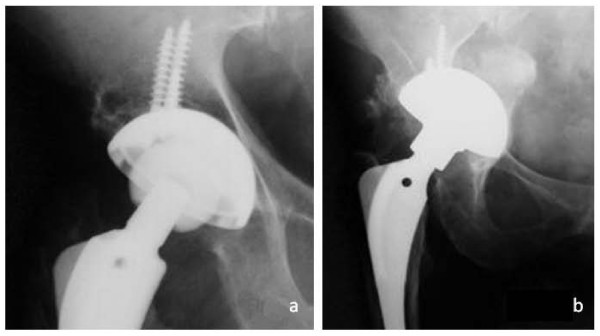
**Radiolucent lines in DeLee zone 1 directly after cup implantation (Plasmacup SC, size 58 mm) without bone graft transplantation (a)**. Complete regression after ten years postoperative (b).

Evidence of radiolucent lines could be confirmed postoperatively as follows:

24% of patients in DeLee zone 1, 6% in zone 2 and 8% in zone 3. Apart from 3 lines in DeLee zone 3, the radiolucent lines regressed in the course of 2 years (Figures 1 and 2). The cups with the 3 remaining radiolucent lines showed no increased migration and the lines did not increase during the observation period. Allogenic bone material was transplanted in 2 of these patients during revision surgery and in one case revision surgery was done without performing bone graft transplantation. Regarding the preoperative acetabular defects, the following data emerged in the 68 post examined cases: 15% Paprosky Type 1, 15% Paprosky Type 2a, 44% Paprosky Type 2b, 7% Paprosky Type 2c, 17% Paprosky Type 3a and 2% Paprosky 3b. There was no correlation found between the Paprosky classification and the clinical results.

In 23 of the 72 cases, intraoperative bone graft transplantation was assessed as necessary. In 14 cases, autogenous bone was used and in 9 cases additional allogenic bone material was used. The autogenous material consisted of ream material obtained during preparation of the acetabulum for cup implantation. In 6 of the 14 cases where the amount of ream material was not sufficient, so that additional cancellous bone in the form of chips was taken from the patient's equilateral iliac crest. The amount of autogenous bone material available for defect replenishment was not sufficient in 9 patients, so that additional allogenic cancellous bone chips from donor femoral heads were used. There was no difference in the clinical result between patients who received intraoperative bone graft transplantation and those whose defects were not intraoperatively replenished with bone.

### Complications

Prosthesis luxation occurred postoperatively in 3 cases (4%). 2 patients were treated conservatively after closed reposition; one patient needed a revision of the femoral head which in retrospect had been chosen too short. One case showed a postoperative femoral paresis which completely regressed after 6 months. A deep venous thrombosis of the leg was detected in 2% of the cases and treated with medication.

Postoperative wound healing disorders occurred in 4 cases (6%) and were revised by surgery. 2 (3%) of these patients underwent a soft tissue revision with wound irrigation.

Another patient was additionally treated with a head and inlay revision during the early postoperative stage. After this, undisturbed healing occurred in these patients.

The worst case scenario was the explantation of the complete prosthesis in one patient because of prosthesis infection performed on 35th postoperative day. 3 soft tissue revisions were performed prior to the explantation. The Girdlestone situation in this patient was left permanently uncorrected.

## Discussion

Achieving good, long term clinical and radiological results even in case of major bone defects is a great challenge for revision total hip arthroplasty. The present study revealed during the observation period of an average of more than eight years, only a minor migration in the first postoperative year in terms of increased inclination movement. Kärrholm et al. as well as Krismer et al. emphasize the high predictive value of early migration regarding aseptic loosening (inclination movement), especially with cementless press-fit-cups [26,27]. The extent of cup migration, which suggests an early loosening is however, assessed differently. While some authors take the view that in primary THA a migration of just 1 mm in the first 2 years considerably reduces the cup's probable lifespan, others state that even a cup migration of 2 mm in the first 2 years only rarely causes aseptic loosening [27,28].

The "Wristing" programme applied for migration analysis in the present study has a specified limit of accuracy of 2 mm or 3.2° [22]. More precise, but incomparably more complex, are the "radiographic stereometry analyses (RSA)" with a limit of accuracy of 0.1 mm and the "single radiograph analysis (EBRA)" with a limit of accuracy of 1 mm [27]. It was not the aim of the present study to observe single cup migration, but to observe migration behaviour of cup types within the entire group over a long period of time. The study design allows for 96% of the patients operated on during the time of survey to be entered into the research and to evaluate radiographs regarding the postoperative course of cup migration. The comparison with prospective studies shows that a follow-up of over 90% over an observation period of more than 8 years can not usually be achieved in prospective studies [29,30]. Despite the retrospective approach, relevant cup migration during the observation period can be ruled out in our study group with a high probability.

The good clinical and functional results achieved in this study are also reported in literature for comparable press-fit-cups of other manufacturers. In 1997 Moskal et al. publicized a study of 31 patients, in which 94% of the cases had good postoperative results after the implantation of cementless press-fit-cups as revision implants [31]. Lachiewicz et al. were able to prove good, to very good, results after the application of press-fit-cups with additional screw fixation as revision cup implants [30,32].

The postoperative points achieved in the Harris-Hip-Score during the aforementioned studies were comparable to those of our study. The Harris-Galante socket used in two of the aforementioned studies is very similar to the one used in the present study. The WOMAC-Score was not applied in the studies mentioned above. The low average of 37.4 points in the WOMAC-Score in our study indicates good postoperative patient satisfaction. 90% of the cases used the possibility of fixing the cup with an additional screw. The benefit of additional screws is unexplained. The decision whether additional screws are used is made by the surgeon during surgery, depending on his view regarding the primary stability achieved. There is no literature containing randomised studies which test their application. Many authors advise using cups as large as possible. Gustke et al. and Obenhaus et al. proved that even major acetabular defects could be reconstructed using large press-fit-cups [33,34]. The definition of the jumbo cup in literature is not clear-cut. According to Patel et al. and Whaley et al., cups are called jumbo cups when their diameter is greater than 65 mm for men and 61 mm for women [35,36]. Ito et al. however, define the jumbo cup using the relative ratio between the size of the implanted acetabular cup and the size of the patient's pelvis [37].

Cementless press-fit-cups with large diameters offer a wide contact area between the acetabular bone and the cup and this is supposed to induce healing. The cup diameters used in our study are, on average, below the mentioned data for jumbo cups. In terms of greater contact between implant and bone, attention was paid to choosing the largest possible cup for every implantation. The migration analysis and radiological results suggest good bone-cup integration. Radiolucent lines in particular, which appeared directly post-operation in 24% of the cases, had become invisible 2 years post-operation.

In our study the acetabular defects were determined according to Paprosky. The allocation of the Paprosky types is comparable to those of other major studies [38]. There is no correlation to be found, neither in literature, nor in this approach, between the classification of the acetabular defect according to Paprosky and the result of the revision surgery. The Paprosky classification seems likely to be more suitable to testing comparability of different studies than to predicting a revision surgery result or influencing the choice of surgical procedure. Elke et al. are even of the opinion that for this purpose the differentiation between "press-fit-suitable" and "press-fit-unsuitable" would be sufficient [39]. The revision situation is defined "press-fit-suitable" when, despite acetabular defects, the press-fit-cup can be fixed to provide lever-out stability.

Literature describes various surgical procedures for the refilling of acetabular defects. This indicates that these defects cannot be reconstructed using a standard method and that the selected method often depends on the individual experience of the surgeon. In the present study, bone material was used in 32% of the cases using allogenic and autogenous bone.

The number of complications in our study is comparable to those of other studies [29,40]. The high death rate of patients, particularly in the postoperative course, expresses the multimorbidity of patients.

A weak point of this retrospective study is the fact that follow-up radiographs were not always available for all patients at the time of examination. This could be made up for through a detailed statistical evaluation of the measured data. A comparison between the pre and postoperative Harris-Hip-Score and WOMAC-Score data is not possible as preoperative scores were not collected. The Harris-Hip-Score and WOMAC-Score data collected postoperatively are, however, within the ranges achieved by other studies. In addition, the small number of patients who, at the time of the follow-up examination, were still regularly taking pain killers and complaints of minor hip pain, document the good result of socket revision surgery using Plasmacup SC.

## Conclusion

The study results support the suitability of the Plasmacup SC press-fit-cup as a secondary cup implant and demonstrate results similar to comparable prostheses of other manufacturers.

The cup can also be used on major medial cup defects. It gains its stability from the contact with the original bone. None of the cups had to be removed because of aseptic loosening.

## Competing interests

The authors declare that they have no competing interests.

## Authors' contributions

SL, GA, TJH, SFW and SMD analyzed and interpreted the patient data regarding the application of the Plasmacup as revision implant. SL, GA and MDS designed the study, collected the data, and contributed to manuscript drafting and critical revision.

GA, SFW and SMD carried out the surgical intervention on the patients. NT performed the statistical analyses. SL was the main composer of the manuscript. All authors read and approved the final manuscript.

## Pre-publication history

The pre-publication history for this paper can be accessed here:

http://www.biomedcentral.com/1471-2474/11/101/prepub

## Supplementary Material

Additional file 1 **Representation of the data determined for cup migration**. The distances are indicated in mm, the inclinations and anteversions in degrees. The indicated p-values always relate to the change compared to the postoperative radiograph. There was no p-value adjustment done for the number of tests conducted. (OPR = top edge of cup, MPR = medial cup edge, PM = cup centre, TF = tear figure). Click here for file
