# Supplementary material for: Results of the cementless Plasmacup in revision total hip arthroplasty: a retrospective study of 72 cases with an average follow-up of eight years
Source: BMC Musculoskelet Disord. 2010 May 27;11:101. doi: 10.1186/1471-2474-11-101 (PMC2887774; doi:10.1186/1471-2474-11-101)
Supplement: Additional file 1 — Representation of the data determined for cup migration. The distances are indicated in mm, the inclinations and anteversions in degrees. The indicated p-values always relate to the change compared to the postoperative radiograph. There was no p-value adjustment done for the number of tests conducted. (OPR = top edge of cup, MPR = medial cup edge, PM = cup centre, TF = tear figure). [file 1471-2474-11-101-S1.DOC]

| **Time of examination** | **OPR – TF longitudinal** | **P-value** | **PM – TF longitudinal** | **p-value** | **MPR – TF transversal** | **p-value** | **PM – TF transversal** | **p-value** | **Inclination** | **p-value** | **Anteversion** | **p-value** |
| --- | --- | --- | --- | --- | --- | --- | --- | --- | --- | --- | --- | --- |
| **postoperative** | 52,44 |  | 22,52 |  | 0,25 |  | 28,87 |  | 28,19 |  | 12,31 |  |
| **0,5 years postoperative** | 51.69 | 0,120 | 22,71 | 0,663 | 0,11 | 0,677 | 28,32 | 0,078 | 30,36 | 0,0005 | 13,63 | 0,022 |
| **1 year postoperative** | 51,82 | 0,323 | 23,17 | 0,241 | -0,28 | 0,222 | 28,14 | 0,070 | 29,43 | 0,1171 | 13,90 | 0,031 |
| **2 to 3 years postoperative** | 52,31 | 0,811 | 23,33 | 0,090 | -0,21 | 0,213 | 28,44 | 0,211 | 30,38 | 0,0041 | 13,10 | 0,211 |
| **4 to 5 years postoperative** | 53,11 | 0,247 | 23,66 | 0,031 | -0,47 | 0,078 | 28,09 | 0,041 | 31,51 | <0.00001 | 13,02 | 0,315 |
| **More than 6 years postoperative** | 52,94 | 0,355 | 23,20 | 0,158 | -0,29 | 0,153 | 28,43 | 0,207 | 31,61 | <0.00001 | 12,86 | 0,392 |
| **Change of position throughout the entire period (ANOVA)** |  | 0.104 |  | 0.229 |  | 0.420 |  | 0.272 |  | <0.0001 |  | 0.181 |

**Table 1:**

Representation of the estimated data determined for cup migration. The distances are indicated in mm, the inclinations and anteversions in degrees. The indicated p-values always relate to the change compared to the postoperative x-ray. There was no p-value adjustment done for the number of tests conducted. (OPR = top edge of cup, MPR = medial cup edge, PM = cup centre, TF = tear figure).
